# Resection and Resolution of Bone Marrow Lesions Associated with an Improvement of Pain after Total Knee Replacement: A Novel Case Study Using a 3-Tesla Metal Artefact Reduction MRI Sequence

**DOI:** 10.1155/2016/6043497

**Published:** 2016-08-25

**Authors:** Thomas Kurien, Robert Kerslake, Brett Haywood, Richard G. Pearson, Brigitte E. Scammell

**Affiliations:** ^1^Arthritis Research UK Pain Centre, Nottingham University, Nottingham, UK; ^2^Academic Orthopaedics, Trauma and Sports Medicine, School of Medicine, The University of Nottingham, Queen's Medical Centre, Derby Road, Nottingham NG7 2UH, UK; ^3^Nottingham University Hospitals NHS Trust, Queen's Medical Centre, Nottingham NG7 2UH, UK; ^4^Academic Radiology, The University of Nottingham, Queen's Medical Centre, Derby Road, Nottingham NG7 2UH, UK

## Abstract

We present our case report using a novel metal artefact reduction magnetic resonance imaging (MRI) sequence to observe resolution of subchondral bone marrow lesions (BMLs), which are strongly associated with pain, in a patient after total knee replacement surgery. Large BMLs were seen preoperatively on the 3-Tesla MRI scans in a patient with severe end stage OA awaiting total knee replacement surgery. Twelve months after surgery, using a novel metal artefact reduction MRI sequence, we were able to visualize the bone-prosthesis interface and found complete resection and resolution of these BMLs. This is the first reported study in the UK to use this metal artefact reduction MRI sequence at 3-Tesla showing that resection and resolution of BMLs in this patient were associated with an improvement of pain and function after total knee replacement surgery. In this case it was associated with a clinically significant improvement of pain and function after surgery. Failure to eradicate these lesions may be a cause of persistent postoperative pain that is seen in up to 20% of patients following TKR surgery.

## 1. Introduction

Painful osteoarthritis (OA) is the 4th largest cause of ill health and disability in the United Kingdom [1]. Subchondral noncystic bone marrow lesions (BMLs) have been identified as key biomarkers in the pathogenesis of osteoarthritis and are characterised as ill-defined areas of low signal intensity compared to normal marrow on T1-weighted images or hyperintense signal change within the subchondral bone on T2-weighted, fat saturated, or short tau inversion recovery MR images [2]. BMLs are not visible on plain radiographs; hence the discovery of these changes on MRI, and their correlation with pain, is of significant clinical interest. Over 75% of patients with painful osteoarthritis of the knee have subchondral BMLs on MRI, and several large studies have reported the correlation of subchondral BMLs with pain [3]. Recent work has also demonstrated that BMLs are a predictor for adjacent cartilage loss in the same knee compartment and that the pain experienced in patients corresponds to the change in size of the BMLs present [4].

Total knee replacement surgery (TKR) is one of the most common orthopaedics procedures performed worldwide and is generally a very successful and cost-effective treatment for improving pain and function in patients with severe painful OA. In the United Kingdom nearly 84,000 primary total knee replacement procedures were performed in 2014.

For many patients, TKR is an effective surgical intervention; however, unfortunately up to 20% of all patients who undergo this surgery report severe dissatisfaction and chronic postoperative pain in spite of objective measures of surgical success [5]. Chronic postoperative pain is defined by the International Association for the Study of Pain (IASP) as pain that is present in a subject six months after surgery. Risk factors associated with the development of chronic postoperative pain after TKR include female gender, lower age, greater preoperative pain, pain at remotes sites, pain catastrophizing, depression, anxiety, and sleep disturbance. The presence of BMLs after TKR has not previously been explored, primarily due to the fact that, even with optimisation of conventional MR sequences, tissues in the immediate vicinity of metallic implants are largely obscured due to image distortion and artefact. However, considerable reduction in artefacts around metallic implants has been achieved using recently introduced MR pulse sequences [6]. We report our early finding using a novel metal artefact reduction MRI sequence showing the complete resection and resolution of BMLs 12 months after TKR surgery with an associated improvement of pain and function of the knee.

## 2. Case Details

**Figure 1:**
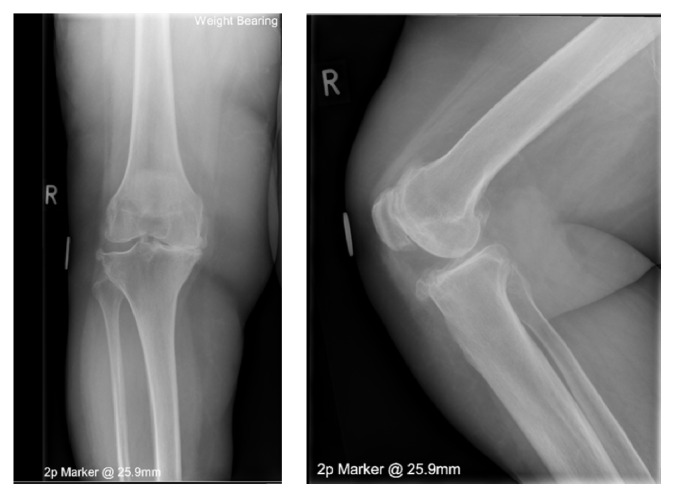
Preoperative weight-bearing AP and lateral radiographs clearly showing tricompartmental knee OA (K-L Grade IV).

The Nottinghamshire Research Ethics Committee gave ethical approval for this patient to receive a metal artefact reduction MRI scan as part of a wider study (REC ref number 10/H0408/115). The patient (female, aged 72, BMI 33.8) awaiting right total knee replacement surgery was recruited after taking informed consent as part of the pilot study. The patient had severe tricompartmental osteoarthritis of the knee with radiographic (Kellegren-Lawrence grade IV) and symptomatic OA prior to the joint replacement surgery (Figure 1). The patient had suffered from progressively worsening symptoms of pain in the right knee for over 15 years. She described pain on standing and on movement as well as disturbance of her normal sleep pattern. On clinical examination she had a varus knee with a mild fixed flexion deformity. Her range of movement was limited to 90° flexion and she was tender on palpation around the medial joint line.

**Figure 2:**
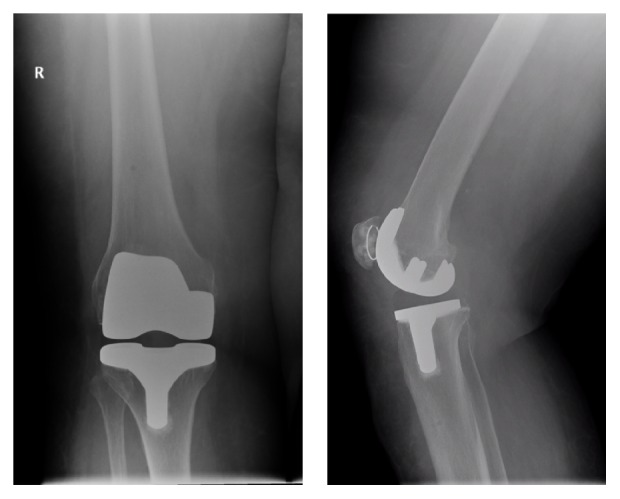
Postoperative AP and lateral radiographs of right Columbus TKR.

Preoperative questionnaires were administered within 2 weeks of the total joint replacement and were repeated 12 months after the operation. The questionnaires included assessment of pain severity, pain catastrophizing, knee function, anxiety, depression, and a measure of the neuropathic pain-like symptoms. The questionnaires included are outlined in Table 1. A navigated Columbus total knee replacement (B. Braun) prosthesis was used as the implant in the case (Figure 2).

## 3. Preoperative Knee MRI Methods

MRI was performed on a 3.0 T system (GE MR 750, GE Healthcare, Waukesha, WI) using an 8-channel phased array transmit-receive knee coil. The knee was scanned in sagittal and axial planes using a proton-density fat-suppressed sequence (2.5 mm thickness, spacing 0.3 mm, repetition time (*T*_*R*_) 3321 ms, effective echo time (*T*_*E*_) 41 ms, echo train length (ETL) 7, and pixel bandwidth (BW) 162) and a coronal T1-weighted sequence (2.5 mm thickness, spacing 0.3 mm, *T*_*R*_ 580 ms, *T*_*E*_ 11 ms, ETL 1, and BW 244).

## 4. Postoperative Knee MRI Methods

Twelve months following knee arthroplasty, the patient was imaged again using the same 3 T MR system and coil. On this occasion, the knee was scanned in sagittal, axial, and coronal planes using an inversion-recovery fat saturated MAVRIC STIR SL sequence (*T*_*R*_/*T*_*E*_ 5000/7.4 ms, inversion time 175 ms, ETL 20, BW 976, slice thickness 4 mm, and zero spacing).

## 5. Bone Marrow Lesion Assessment

Subchondral BMLs (defined as areas of altered subchondral marrow signal adjacent to the underlying the medial tibia, medial femoral, lateral tibia, lateral femoral, and patellar aspects of the knee joint) were assessed quantitatively using OSIRIX software (University of Geneva, Geneva, Switzerland). A single trained observer measured the maximum area (cm^2^) of the BMLs preoperatively and twelve months postoperatively by manually selecting the MRI slice with the greatest BML size. The MRIs at both time points were read but the observer was blinded to the questionnaire results of the patient.

## 6. Results

**Figure 3:**
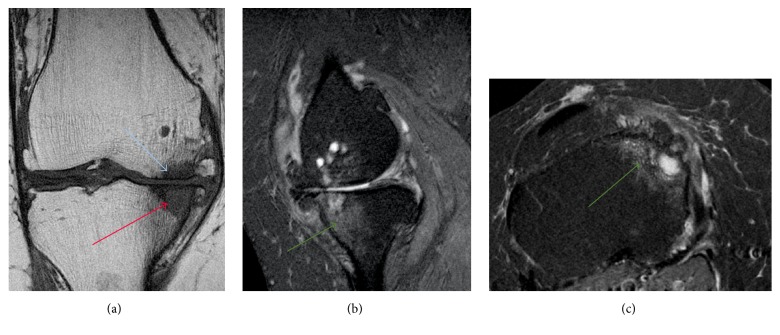
(a) Coronal T1 MRI with contrast image in painful knee OA patient showing large medial proximal tibial BML (red arrow) and smaller medial distal femoral BML (blue arrow). (b) Corresponding proton density weighted fat suppressed sagittal MRI sequence showing the large anteromedial BML (green arrow). (c) Axial proton density weighted fat suppressed MRI showing the tibia BML.

Using the 3-Tesla preoperative knee MRI, we were able to visualise a large anteromedial BML (2.21 cm^2^) in a patient with painful knee osteoarthritis. There was also a corresponding smaller BML in the posterior-medial distal femur (1.17 cm^2^). The preoperative MRI images with the BMLs are shown in Figure 3.

**Figure 4:**
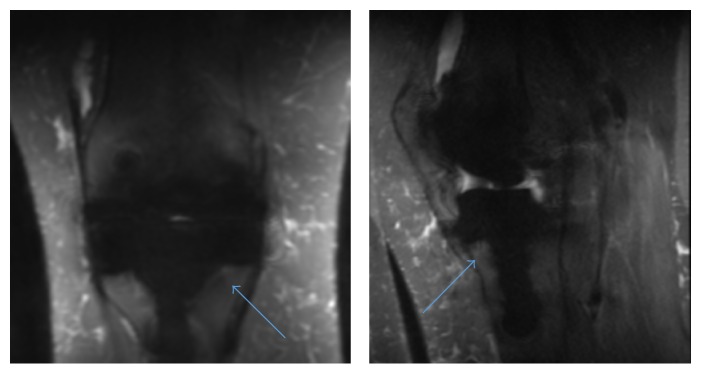
Coronal and Sagittal MAVRIC® STIR SLR MRI showing complete resection and resolution of BMLs 12 months after TKR surgery (blue arrows).

Using the MAVRIC metal artefact reduction MRI sequence 12 months postoperatively, we noted complete resection or resolution of the BMLs seen preoperatively; with the exception of a narrow band of artefact in the immediate vicinity of the implants, the subchondral marrow signal could be clearly visualised on postoperative images and was seen to have returned to normal. Of particular note, the largest preoperative BML that extended 2.6 cm caudal to the medial tibial plateau had been resected or resolved in its entirety and this was accompanied by an improvement in the patient's reported pain (Figure 4 and Table 2).

## 7. Discussion

This is the first reported use of the novel MAVRIC MRI sequence at 3-Tesla used in the UK after TKR surgery to report the relationship between resolving pain and the disappearance of BMLs. Our case study has reported that the resection and resolution of these nociceptive BMLs are associated with an improvement of pain and function after TKR surgery as seen with the VAS and PainDetect® score as well as the Oxford Knee Score. The patient recruited as part of this pilot work demonstrated neuropathic pain-like symptoms preoperatively as observed by her high PainDetect score. Depression and anxiety scores improved postoperatively but there was small worsening in the catastrophizing score after surgery. During TKR, approximately 10 mm of proximal femur and 10 mm of proximal tibia were resected; this included part but not all of the BMLs that had been documented. However, 12 months after arthroplasty imaging showed complete resolution of the remainder of the BML. The postoperative low PainDetect score is indicative of resolution of the neuropathic pain-like symptoms seen in central sensitization. Pain in knee osteoarthritis can originate from many sources including the subchondral bone as bone marrow lesions, synovitis, the periosteum, and osteophytes and there are many factors that can contribute to postoperative pain. However, through this important case report and future research, we hypothesise that failure to fully resect these lesions at the time of TKR surgery or the development of new BMLs within the subchondral bone after TKR surgery may act as a continued peripheral nociceptive input, maintaining pain, altered central processing, and central sensitization after TKR. This may explain some of the variance in pain relief after TKR. Further investigation into these lesions is required to understand the role of BMLs in chronic postoperative pain.

## Figures and Tables

**Table 1:** Pre- and postoperative questionnaires.

Assessment	Questionnaire	Comments
Pain severity	Visual Analogue Scale (VAS)	Used to categorise individuals with high or low pain. Scores range from 0 to 100, with score ≥ 60 representing high pain intensity.

Pain catastrophizing	Pain Catastrophizing Scale (PCS)	This 13-item scale uses questions about coping with pain. Scores range from 0 to 52 with a higher score seen in individuals that are high catastrophizers.

Depression	Beck Depression Inventory (BDI)	21-question assessment rating inventory attitudes and symptoms of depression. The score ranges from 0 to 63 with a score ≥ 19 indicative of clinical depression.

Anxiety	State-Trait Anxiety (STAI)	Assesses how patients feel and reflects situational factors, which influence anxiety levels. Scores range from 20 to 80 with higher scores associated with higher anxiety.

Neuropathic pain-like symptoms	PainDetect	Individuals are categorised as having neuropathic pain based on the results of a joint-specific version of the PainDetect questionnaire. Scores range from 0 to 39 with a score > 12 indicating possible neuropathic pain and a score ≥ 19 indicating likely neuropathic pain.

Pain and function of the knee	Oxford Knee Score (OKS)	A 12-item questionnaire that assesses pain and function of the knee. Scores range from 0 to 48 with a lower score indicating severe knee arthritis or pain and reduced function in the knee. A higher score between 40 and 48 indicated satisfactory joint function. A minimally clinically important difference of 4 points on the scale can be used to assess the results of an intervention, for example, pain relief or TKR surgery.

**Table 2:** Pre- and post-TKR questionnaire results.

Questionnaire	Pre-op score	Postoperative score
Visual Analogue Scale (VAS)	80/100	0/100
PainDetect	20 (neuropathic)	8
Beck Depression Inventory	14	8
State-Trait Anxiety	51	38
Pain Catastrophizing Scale	34	38
Oxford Knee Score	9	43
